# (*E*)-2-[(1-Benzyl­piperidin-4-yl)imino­meth­yl]phenol

**DOI:** 10.1107/S160053681105197X

**Published:** 2011-12-10

**Authors:** Rui-Qin Fang, Zhu-Ping Xiao, Yun Zuo

**Affiliations:** a State Key Laboratory of Pharmaceutical Biotechnology, Nanjing University, Nanjing 210093, People’s Republic of China; b School of Life Science and Technology, University of Electronic Science and Technology of China, Chengdu 610054, People’s Republic of China

## Abstract

There are two mol­ecules in the asymmetric unit of the title compound, C_19_H_22_N_2_O. Both mol­ecules have an *E* conformation about their C=N bonds and both piperdine rings adopt chair conformations with their N atoms adopting pyramidal geometries [bond angle sums = 329.8 (4) and 330.2 (4)°]. Both mol­ecules feature an intra­molecular O—H⋯N hydrogen bond, which generates an *S*(6) ring. The dihedral angles between the phenyl and benzene ring planes are 45.97 (18) and 66.0 (2)°. Short O—H⋯O contacts occur in the crystal.

## Related literature

For a related structure, see: Stilinovic *et al.* (2008 ▶).
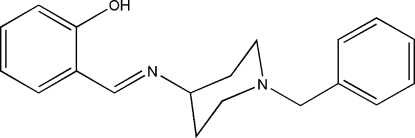


## Experimental

### 

#### Crystal data

C_19_H_22_N_2_O
*M* *_r_* = 294.39
Monoclinic, 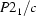

*a* = 10.603 (2) Å
*b* = 9.6330 (19) Å
*c* = 32.595 (7) Å
β = 95.60 (3)°
*V* = 3313.3 (11) Å^3^
*Z* = 8
Mo *K*α radiation
μ = 0.07 mm^−1^
*T* = 293 K
0.40 × 0.40 × 0.20 mm

#### Data collection

Enraf–Nonius CAD-4 diffractometer
Absorption correction: ψ scan (North *et al.*, 1968 ▶) *T*_min_ = 0.971, *T*_max_ = 0.986
6837 measured reflections
6473 independent reflections
2764 reflections with *I* > 2σ(*I*)
*R*_int_ = 0.117
3 standard reflections every 200 reflections  intensity decay: 1%

#### Refinement

*R*[*F* ^2^ > 2σ(*F* ^2^)] = 0.082
*wR*(*F* ^2^) = 0.268
*S* = 1.09
6473 reflections
406 parameters
H atoms treated by a mixture of independent and constrained refinement
Δρ_max_ = 0.22 e Å^−3^
Δρ_min_ = −0.20 e Å^−3^

### 

Data collection: *CAD-4 Software* (Enraf–Nonius, 1989 ▶); cell refinement: *CAD-4 Software*; data reduction: *XCAD4* (Harms & Wocadlo, 1995 ▶); program(s) used to solve structure: *SHELXS97* (Sheldrick, 2008 ▶); program(s) used to refine structure: *SHELXL97* (Sheldrick, 2008 ▶); molecular graphics: *SHELXTL* (Sheldrick, 2008 ▶); software used to prepare material for publication: *SHELXTL*.

## Supplementary Material

Crystal structure: contains datablock(s) global, I. DOI: 10.1107/S160053681105197X/hb6532sup1.cif

Structure factors: contains datablock(s) I. DOI: 10.1107/S160053681105197X/hb6532Isup2.hkl

Supplementary material file. DOI: 10.1107/S160053681105197X/hb6532Isup3.cdx

Supplementary material file. DOI: 10.1107/S160053681105197X/hb6532Isup4.cml

Additional supplementary materials:  crystallographic information; 3D view; checkCIF report

## Figures and Tables

**Table 1:** Hydrogen-bond geometry (Å, °)

*D*—H⋯*A*	*D*—H	H⋯*A*	*D*⋯*A*	*D*—H⋯*A*
O1—H1⋯N1	1.01 (8)	1.73 (7)	2.597 (5)	141 (6)
O2—H2*A*⋯N3	1.05 (7)	1.66 (7)	2.588 (6)	144 (5)
O1—H1⋯O1^i^	1.01 (8)	2.49 (7)	2.869 (7)	102 (5)

Symmetry code: (i) 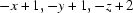.

**Fig. 1.**
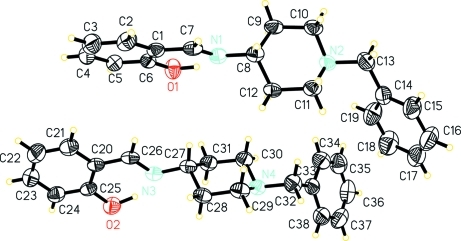
The structure of (I) showing 35% probability displacement ellipsoids.

**Fig. 2.**
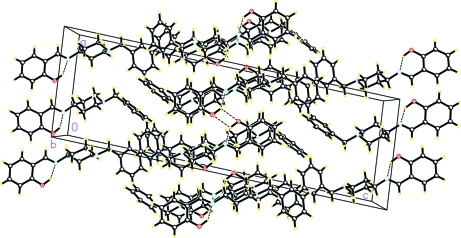
The crystal packing of (I), viewed along the b axis. Hydrogen bonds are shown as dashed lines.

## supplementary crystallographic information

### Comment

The cystal structure of 1,4-bis-((1-benzylpiperidin-4-ylimino)methyl)benzene has been reported, which was synthesized by 4-amino-*N*-benzylpiperidine and terephtaldialdehyde. (Stilinovic *et al.*, 2008). While, the title compund has been obtained by 4-amino-*N*-benzylpiperidine and salicyaldehyde. The molecular structure of title compound (I) with atom numbering are given in is shown in Fig. 1, there are two (*E*)-2-((1-benzylpiperidin-4-ylimino)methyl)phenol in an asymmetric unit. Both C7=N1 and C26=N3 are of the E configuration, with the bond lengths of 1.262 (6) and 1.267 (6) Å. The torsion angle of C9—C8—N1—C7 and C28—C27—N3—C26 is -118.2 (5) ° and 107.9 (5) °, respectively. The Rms of two six-member piperidine rings of chair conformation are 0.2354 Å and 0.2322 Å. The dihedral angles between two phenyl planes in two molecules are 45.97 (18) and 65.97 (21)°.  In each molecule, intramolecular O—H···N hydrogen bonds occur, and molecules are linked through intermolecular O—H···O hydrogen bonds to form a packing network along *b* axis.(Fig. 2).

### Experimental

The title compound was prepared by stirring a mixture of salicylaldehyde (122 mg, 1 mmol) and 4-amino-*N*-benzylpiperidine (190 mg, 1 mmol) in methanol (15 ml) for 4 h at room temperature. After keeping the solution in air for 3 d, yellow block-shaped crystals of (I) were formed. The crystals were isolated, washed three times with methanol and dried in a vacuum desiccator containing anhydrous CaCl_2_.

### Refinement

All the H atoms, were placed in idealized positions (C—H = 0.93- 0.96 Å, O—H = 0.82 Å) and refined as riding with *U*_iso_(H) = 1.2*U*_eq_(C) and *U*_iso_(H) = 1.5*U*_eq_(O).

### Crystal data

**Table:**

C_19_H_22_N_2_O	*F*(000) = 1264
*M**_r_* = 294.39	*D*_x_ = 1.180 Mg m^−^^3^
Monoclinic, *P*2_1_/*c*	Mo *K*α radiation, λ = 0.71073 Å
Hall symbol: -P 2ybc	Cell parameters from 2318 reflections
*a* = 10.603 (2) Å	θ = 2.6–24.7°
*b* = 9.6330 (19) Å	µ = 0.07 mm^−^^1^
*c* = 32.595 (7) Å	*T* = 293 K
β = 95.60 (3)°	Block, yellow
*V* = 3313.3 (11) Å^3^	0.40 × 0.40 × 0.20 mm
*Z* = 8	

### Data collection

**Table:**

Enraf–Nonius CAD-4 diffractometer	2764 reflections with *I* > 2σ(*I*)
Radiation source: fine-focus sealed tube	*R*_int_ = 0.117
graphite	θ_max_ = 26.0°, θ_min_ = 1.3°
ω/2θ scan	*h* = 0→13
Absorption correction: ψ scan (North *et al.*, 1968)	*k* = 0→11
*T*_min_ = 0.971, *T*_max_ = 0.986	*l* = −40→39
6837 measured reflections	3 standard reflections every 200 reflections
6473 independent reflections	intensity decay: 1%

### Refinement

**Table:**

Refinement on *F*^2^	Secondary atom site location: difference Fourier map
Least-squares matrix: full	Hydrogen site location: inferred from neighbouring sites
*R*[*F*^2^ > 2σ(*F*^2^)] = 0.082	H atoms treated by a mixture of independent and constrained refinement
*wR*(*F*^2^) = 0.268	*w* = 1/[σ^2^(*F*_o_^2^) + (0.0628*P*)^2^ + 4.8871*P*] where *P* = (*F*_o_^2^ + 2*F*_c_^2^)/3
*S* = 1.09	(Δ/σ)_max_ < 0.001
6473 reflections	Δρ_max_ = 0.22 e Å^−^^3^
406 parameters	Δρ_min_ = −0.20 e Å^−^^3^
0 restraints	Extinction correction: *SHELXL97* (Sheldrick, 2008), Fc^*^=kFc[1+0.001xFc^2^λ^3^/sin(2θ)]^-1/4^
Primary atom site location: structure-invariant direct methods	Extinction coefficient: 0.0089 (11)

### Special details

**Table:**

Geometry. All e.s.d.'s (except the e.s.d. in the dihedral angle between two l.s. planes) are estimated using the full covariance matrix. The cell e.s.d.'s are taken into account individually in the estimation of e.s.d.'s in distances, angles and torsion angles; correlations between e.s.d.'s in cell parameters are only used when they are defined by crystal symmetry. An approximate (isotropic) treatment of cell e.s.d.'s is used for estimating e.s.d.'s involving l.s. planes.
Refinement. Refinement of *F*^2^ against ALL reflections. The weighted *R*-factor *wR* and goodness of fit *S* are based on *F*^2^, conventional *R*-factors *R* are based on *F*, with *F* set to zero for negative *F*^2^. The threshold expression of *F*^2^ > σ(*F*^2^) is used only for calculating *R*-factors(gt) *etc*. and is not relevant to the choice of reflections for refinement. *R*-factors based on *F*^2^ are statistically about twice as large as those based on *F*, and *R*- factors based on ALL data will be even larger.

### Fractional atomic coordinates and isotropic or equivalent isotropic displacement parameters (Å^2^)

**Table:**

	*x*	*y*	*z*	*U*_iso_*/*U*_eq_	
C1	0.3352 (5)	0.6495 (5)	1.08004 (15)	0.0556 (13)	
C2	0.3051 (6)	0.6876 (5)	1.11893 (17)	0.0748 (16)	
H2	0.2226	0.7148	1.1223	0.090*	
C3	0.3932 (8)	0.6864 (6)	1.15252 (19)	0.088 (2)	
H3	0.3714	0.7136	1.1783	0.106*	
C4	0.5159 (7)	0.6437 (6)	1.14746 (18)	0.0829 (18)	
H4	0.5767	0.6431	1.1700	0.099*	
C5	0.5483 (5)	0.6028 (6)	1.10994 (16)	0.0698 (15)	
H5	0.6304	0.5726	1.1072	0.084*	
C6	0.4601 (5)	0.6057 (5)	1.07583 (15)	0.0581 (13)	
C7	0.2416 (5)	0.6562 (5)	1.04446 (17)	0.0576 (13)	
H7	0.1604	0.6872	1.0482	0.069*	
C8	0.1693 (5)	0.6312 (5)	0.97399 (15)	0.0566 (13)	
H8	0.0912	0.6681	0.9836	0.068*	
C9	0.1432 (5)	0.4880 (5)	0.95584 (16)	0.0632 (14)	
H9A	0.2228	0.4443	0.9510	0.076*	
H9B	0.1031	0.4313	0.9755	0.076*	
C10	0.0583 (5)	0.4942 (5)	0.91573 (16)	0.0672 (15)	
H10A	0.0483	0.4015	0.9043	0.081*	
H10B	−0.0248	0.5275	0.9212	0.081*	
C11	0.1222 (5)	0.7256 (5)	0.90270 (16)	0.0621 (14)	
H11A	0.0398	0.7586	0.9090	0.075*	
H11B	0.1527	0.7876	0.8824	0.075*	
C12	0.2130 (5)	0.7276 (5)	0.94143 (15)	0.0601 (14)	
H12A	0.2195	0.8214	0.9522	0.072*	
H12B	0.2965	0.6993	0.9348	0.072*	
C13	0.0265 (5)	0.5833 (6)	0.84734 (17)	0.0780 (17)	
H13A	−0.0563	0.6171	0.8529	0.094*	
H13B	0.0164	0.4879	0.8381	0.094*	
C14	0.0717 (5)	0.6680 (6)	0.81311 (17)	0.0674 (15)	
C15	−0.0101 (6)	0.7503 (7)	0.7894 (2)	0.094 (2)	
H15	−0.0943	0.7548	0.7950	0.113*	
C16	0.0286 (8)	0.8274 (8)	0.7570 (2)	0.112 (3)	
H16	−0.0295	0.8827	0.7413	0.134*	
C17	0.1515 (8)	0.8224 (8)	0.74818 (19)	0.100 (2)	
H17	0.1779	0.8738	0.7264	0.121*	
C18	0.2346 (7)	0.7413 (9)	0.7715 (2)	0.114 (3)	
H18	0.3188	0.7375	0.7659	0.137*	
C19	0.1952 (7)	0.6646 (8)	0.8034 (2)	0.095 (2)	
H19	0.2535	0.6088	0.8189	0.114*	
C20	0.8513 (5)	0.8847 (5)	1.08785 (16)	0.0553 (13)	
C21	0.8191 (6)	0.8677 (6)	1.12796 (18)	0.0803 (17)	
H21	0.7370	0.8410	1.1321	0.096*	
C22	0.9048 (7)	0.8893 (8)	1.1613 (2)	0.100 (2)	
H22	0.8815	0.8767	1.1878	0.120*	
C23	1.0274 (6)	0.9302 (6)	1.15529 (19)	0.0836 (18)	
H23	1.0865	0.9447	1.1779	0.100*	
C24	1.0607 (5)	0.9488 (6)	1.11692 (18)	0.0693 (15)	
H24	1.1427	0.9772	1.1134	0.083*	
C25	0.9754 (5)	0.9267 (5)	1.08249 (16)	0.0564 (13)	
C26	0.7581 (5)	0.8606 (5)	1.05304 (16)	0.0573 (13)	
H26	0.6773	0.8313	1.0578	0.069*	
C27	0.6858 (5)	0.8565 (5)	0.98220 (15)	0.0605 (14)	
H27	0.6128	0.8100	0.9924	0.073*	
C28	0.6448 (5)	0.9970 (5)	0.96375 (16)	0.0632 (14)	
H28A	0.6056	1.0515	0.9841	0.076*	
H28B	0.7185	1.0472	0.9563	0.076*	
C29	0.5523 (5)	0.9782 (5)	0.92612 (17)	0.0661 (15)	
H29A	0.4767	0.9324	0.9339	0.079*	
H29B	0.5278	1.0684	0.9148	0.079*	
C30	0.6422 (5)	0.7581 (5)	0.91122 (16)	0.0694 (15)	
H30A	0.6789	0.7040	0.8903	0.083*	
H30B	0.5669	0.7102	0.9183	0.083*	
C31	0.7362 (5)	0.7691 (5)	0.94894 (16)	0.0682 (15)	
H31A	0.8142	0.8099	0.9413	0.082*	
H31B	0.7557	0.6768	0.9597	0.082*	
C32	0.5155 (5)	0.8848 (6)	0.85825 (17)	0.0768 (16)	
H32A	0.4821	0.9766	0.8514	0.092*	
H32B	0.4453	0.8272	0.8650	0.092*	
C33	0.5680 (5)	0.8258 (6)	0.82132 (17)	0.0676 (15)	
C34	0.5566 (6)	0.6876 (7)	0.8109 (2)	0.0862 (18)	
H34	0.5168	0.6284	0.8281	0.103*	
C35	0.6009 (7)	0.6343 (8)	0.7766 (2)	0.099 (2)	
H35	0.5930	0.5398	0.7711	0.119*	
C36	0.6567 (7)	0.7187 (10)	0.7504 (2)	0.102 (2)	
H36	0.6847	0.6827	0.7264	0.122*	
C37	0.6717 (7)	0.8574 (9)	0.7594 (2)	0.103 (2)	
H37	0.7110	0.9157	0.7417	0.123*	
C38	0.6278 (6)	0.9096 (7)	0.7950 (2)	0.0878 (19)	
H38	0.6390	1.0032	0.8013	0.105*	
N1	0.2660 (4)	0.6217 (4)	1.00875 (13)	0.0562 (11)	
N2	0.1097 (4)	0.5853 (4)	0.88561 (12)	0.0586 (11)	
N3	0.7836 (4)	0.8785 (4)	1.01623 (13)	0.0578 (11)	
N4	0.6071 (4)	0.8956 (4)	0.89468 (13)	0.0597 (11)	
O1	0.4961 (4)	0.5668 (4)	1.03919 (12)	0.0746 (11)	
O2	1.0116 (4)	0.9462 (4)	1.04478 (12)	0.0777 (12)	
H2A	0.937 (7)	0.915 (7)	1.023 (2)	0.14 (3)*	
H1	0.424 (7)	0.574 (8)	1.017 (2)	0.15 (3)*	

### Atomic displacement parameters (Å^2^)

**Table:**

	*U*^11^	*U*^22^	*U*^33^	*U*^12^	*U*^13^	*U*^23^
C1	0.072 (4)	0.037 (3)	0.060 (3)	−0.002 (2)	0.016 (3)	−0.001 (2)
C2	0.102 (5)	0.061 (4)	0.065 (4)	0.009 (3)	0.024 (4)	−0.001 (3)
C3	0.133 (6)	0.067 (4)	0.066 (4)	−0.003 (4)	0.019 (4)	−0.013 (3)
C4	0.109 (5)	0.075 (4)	0.062 (4)	−0.018 (4)	−0.009 (4)	0.003 (3)
C5	0.082 (4)	0.068 (4)	0.059 (3)	−0.013 (3)	0.003 (3)	0.004 (3)
C6	0.074 (4)	0.047 (3)	0.054 (3)	−0.011 (3)	0.007 (3)	−0.003 (2)
C7	0.058 (3)	0.039 (3)	0.078 (4)	0.001 (2)	0.016 (3)	0.007 (3)
C8	0.054 (3)	0.047 (3)	0.069 (3)	0.007 (2)	0.008 (3)	0.005 (3)
C9	0.063 (3)	0.041 (3)	0.086 (4)	−0.002 (2)	0.007 (3)	0.010 (3)
C10	0.066 (3)	0.047 (3)	0.087 (4)	−0.005 (3)	0.000 (3)	0.008 (3)
C11	0.069 (3)	0.041 (3)	0.077 (4)	0.004 (3)	0.004 (3)	0.006 (3)
C12	0.073 (4)	0.036 (3)	0.071 (3)	−0.007 (2)	0.004 (3)	0.000 (2)
C13	0.075 (4)	0.069 (4)	0.086 (4)	−0.009 (3)	−0.011 (3)	−0.005 (3)
C14	0.067 (4)	0.066 (4)	0.066 (3)	0.004 (3)	−0.005 (3)	−0.005 (3)
C15	0.070 (4)	0.114 (6)	0.094 (5)	0.011 (4)	−0.010 (4)	0.017 (4)
C16	0.114 (7)	0.134 (7)	0.084 (5)	0.015 (5)	−0.007 (4)	0.035 (5)
C17	0.104 (6)	0.129 (6)	0.067 (4)	−0.009 (5)	0.003 (4)	0.009 (4)
C18	0.085 (5)	0.163 (8)	0.094 (5)	0.009 (5)	0.011 (4)	0.012 (5)
C19	0.088 (5)	0.115 (6)	0.082 (4)	0.028 (4)	0.003 (4)	0.015 (4)
C20	0.060 (3)	0.040 (3)	0.068 (3)	0.003 (2)	0.014 (3)	0.005 (2)
C21	0.074 (4)	0.091 (4)	0.079 (4)	−0.013 (3)	0.020 (3)	0.005 (4)
C22	0.107 (6)	0.122 (6)	0.072 (4)	−0.023 (5)	0.014 (4)	0.010 (4)
C23	0.092 (5)	0.087 (5)	0.070 (4)	−0.002 (4)	−0.002 (3)	0.005 (3)
C24	0.064 (4)	0.065 (4)	0.078 (4)	0.008 (3)	0.005 (3)	0.013 (3)
C25	0.061 (3)	0.045 (3)	0.065 (3)	0.008 (2)	0.014 (3)	0.009 (2)
C26	0.060 (3)	0.038 (3)	0.077 (4)	−0.002 (2)	0.022 (3)	0.003 (3)
C27	0.066 (3)	0.047 (3)	0.070 (3)	−0.009 (3)	0.014 (3)	0.002 (3)
C28	0.062 (3)	0.047 (3)	0.081 (4)	0.002 (3)	0.013 (3)	−0.004 (3)
C29	0.058 (3)	0.049 (3)	0.093 (4)	0.009 (3)	0.014 (3)	0.002 (3)
C30	0.085 (4)	0.049 (3)	0.075 (4)	0.005 (3)	0.007 (3)	−0.005 (3)
C31	0.084 (4)	0.043 (3)	0.077 (4)	0.011 (3)	0.007 (3)	0.002 (3)
C32	0.065 (4)	0.076 (4)	0.088 (4)	0.010 (3)	−0.001 (3)	0.001 (3)
C33	0.064 (4)	0.063 (4)	0.073 (4)	0.003 (3)	−0.009 (3)	0.003 (3)
C34	0.089 (5)	0.076 (4)	0.094 (5)	−0.003 (4)	0.008 (4)	−0.008 (4)
C35	0.103 (6)	0.096 (5)	0.095 (5)	0.012 (4)	−0.004 (4)	−0.013 (5)
C36	0.092 (5)	0.140 (7)	0.070 (4)	0.032 (5)	−0.005 (4)	−0.012 (5)
C37	0.106 (6)	0.123 (7)	0.078 (5)	0.008 (5)	0.003 (4)	0.028 (5)
C38	0.092 (5)	0.076 (4)	0.091 (5)	0.002 (4)	−0.013 (4)	0.007 (4)
N1	0.058 (3)	0.049 (2)	0.063 (3)	0.004 (2)	0.010 (2)	0.002 (2)
N2	0.065 (3)	0.041 (2)	0.068 (3)	−0.004 (2)	−0.005 (2)	0.000 (2)
N3	0.058 (3)	0.049 (2)	0.069 (3)	−0.003 (2)	0.016 (2)	0.000 (2)
N4	0.056 (3)	0.047 (2)	0.075 (3)	0.007 (2)	0.004 (2)	0.001 (2)
O1	0.065 (2)	0.101 (3)	0.059 (2)	0.006 (2)	0.010 (2)	−0.011 (2)
O2	0.062 (2)	0.101 (3)	0.072 (3)	−0.010 (2)	0.019 (2)	0.008 (2)

### Geometric parameters (Å, °)

**Table:**

C1—C2	1.386 (7)	C20—C25	1.404 (6)
C1—C6	1.409 (7)	C20—C26	1.449 (7)
C1—C7	1.453 (7)	C21—C22	1.362 (8)
C2—C3	1.368 (8)	C21—H21	0.9300
C2—H2	0.9300	C22—C23	1.390 (8)
C3—C4	1.389 (8)	C22—H22	0.9300
C3—H3	0.9300	C23—C24	1.344 (7)
C4—C5	1.361 (7)	C23—H23	0.9300
C4—H4	0.9300	C24—C25	1.387 (7)
C5—C6	1.381 (7)	C24—H24	0.9300
C5—H5	0.9300	C25—O2	1.336 (6)
C6—O1	1.342 (6)	C26—N3	1.267 (6)
C7—N1	1.262 (6)	C26—H26	0.9300
C7—H7	0.9300	C27—N3	1.458 (6)
C8—N1	1.455 (6)	C27—C31	1.511 (7)
C8—C9	1.516 (7)	C27—C28	1.527 (7)
C8—C12	1.517 (6)	C27—H27	0.9800
C8—H8	0.9800	C28—C29	1.504 (7)
C9—C10	1.514 (7)	C28—H28A	0.9700
C9—H9A	0.9700	C28—H28B	0.9700
C9—H9B	0.9700	C29—N4	1.462 (6)
C10—N2	1.461 (6)	C29—H29A	0.9700
C10—H10A	0.9700	C29—H29B	0.9700
C10—H10B	0.9700	C30—N4	1.464 (6)
C11—N2	1.462 (6)	C30—C31	1.509 (7)
C11—C12	1.512 (6)	C30—H30A	0.9700
C11—H11A	0.9700	C30—H30B	0.9700
C11—H11B	0.9700	C31—H31A	0.9700
C12—H12A	0.9700	C31—H31B	0.9700
C12—H12B	0.9700	C32—N4	1.463 (6)
C13—N2	1.456 (6)	C32—C33	1.487 (7)
C13—C14	1.498 (7)	C32—H32A	0.9700
C13—H13A	0.9700	C32—H32B	0.9700
C13—H13B	0.9700	C33—C38	1.376 (8)
C14—C15	1.360 (7)	C33—C34	1.377 (8)
C14—C19	1.377 (8)	C34—C35	1.353 (8)
C15—C16	1.384 (9)	C34—H34	0.9300
C15—H15	0.9300	C35—C36	1.357 (9)
C16—C17	1.363 (9)	C35—H35	0.9300
C16—H16	0.9300	C36—C37	1.374 (10)
C17—C18	1.355 (9)	C36—H36	0.9300
C17—H17	0.9300	C37—C38	1.388 (9)
C18—C19	1.372 (9)	C37—H37	0.9300
C18—H18	0.9300	C38—H38	0.9300
C19—H19	0.9300	O1—H1	1.01 (8)
C20—C21	1.393 (7)	O2—H2A	1.05 (7)
			
C2—C1—C6	118.1 (5)	C20—C21—H21	119.2
C2—C1—C7	121.2 (5)	C21—C22—C23	119.4 (6)
C6—C1—C7	120.7 (5)	C21—C22—H22	120.3
C3—C2—C1	121.9 (6)	C23—C22—H22	120.3
C3—C2—H2	119.0	C24—C23—C22	120.2 (6)
C1—C2—H2	119.0	C24—C23—H23	119.9
C2—C3—C4	118.8 (6)	C22—C23—H23	119.9
C2—C3—H3	120.6	C23—C24—C25	121.5 (6)
C4—C3—H3	120.6	C23—C24—H24	119.2
C5—C4—C3	120.9 (6)	C25—C24—H24	119.2
C5—C4—H4	119.5	O2—C25—C24	120.0 (5)
C3—C4—H4	119.5	O2—C25—C20	120.8 (5)
C4—C5—C6	120.5 (6)	C24—C25—C20	119.2 (5)
C4—C5—H5	119.8	N3—C26—C20	121.8 (5)
C6—C5—H5	119.8	N3—C26—H26	119.1
O1—C6—C5	118.8 (5)	C20—C26—H26	119.1
O1—C6—C1	121.4 (5)	N3—C27—C31	110.5 (4)
C5—C6—C1	119.7 (5)	N3—C27—C28	109.0 (4)
N1—C7—C1	122.6 (5)	C31—C27—C28	108.6 (4)
N1—C7—H7	118.7	N3—C27—H27	109.6
C1—C7—H7	118.7	C31—C27—H27	109.6
N1—C8—C9	109.6 (4)	C28—C27—H27	109.6
N1—C8—C12	110.0 (4)	C29—C28—C27	110.6 (4)
C9—C8—C12	109.9 (4)	C29—C28—H28A	109.5
N1—C8—H8	109.1	C27—C28—H28A	109.5
C9—C8—H8	109.1	C29—C28—H28B	109.5
C12—C8—H8	109.1	C27—C28—H28B	109.5
C10—C9—C8	111.8 (4)	H28A—C28—H28B	108.1
C10—C9—H9A	109.3	N4—C29—C28	111.4 (4)
C8—C9—H9A	109.3	N4—C29—H29A	109.3
C10—C9—H9B	109.3	C28—C29—H29A	109.3
C8—C9—H9B	109.3	N4—C29—H29B	109.3
H9A—C9—H9B	107.9	C28—C29—H29B	109.3
N2—C10—C9	112.0 (4)	H29A—C29—H29B	108.0
N2—C10—H10A	109.2	N4—C30—C31	111.1 (4)
C9—C10—H10A	109.2	N4—C30—H30A	109.4
N2—C10—H10B	109.2	C31—C30—H30A	109.4
C9—C10—H10B	109.2	N4—C30—H30B	109.4
H10A—C10—H10B	107.9	C31—C30—H30B	109.4
N2—C11—C12	110.9 (4)	H30A—C30—H30B	108.0
N2—C11—H11A	109.5	C30—C31—C27	111.8 (4)
C12—C11—H11A	109.5	C30—C31—H31A	109.3
N2—C11—H11B	109.5	C27—C31—H31A	109.3
C12—C11—H11B	109.5	C30—C31—H31B	109.3
H11A—C11—H11B	108.1	C27—C31—H31B	109.3
C11—C12—C8	111.3 (4)	H31A—C31—H31B	107.9
C11—C12—H12A	109.4	N4—C32—C33	114.4 (4)
C8—C12—H12A	109.4	N4—C32—H32A	108.7
C11—C12—H12B	109.4	C33—C32—H32A	108.7
C8—C12—H12B	109.4	N4—C32—H32B	108.7
H12A—C12—H12B	108.0	C33—C32—H32B	108.7
N2—C13—C14	114.8 (5)	H32A—C32—H32B	107.6
N2—C13—H13A	108.6	C38—C33—C34	116.6 (6)
C14—C13—H13A	108.6	C38—C33—C32	120.8 (6)
N2—C13—H13B	108.6	C34—C33—C32	122.6 (6)
C14—C13—H13B	108.6	C35—C34—C33	122.8 (7)
H13A—C13—H13B	107.5	C35—C34—H34	118.6
C15—C14—C19	116.8 (6)	C33—C34—H34	118.6
C15—C14—C13	120.6 (6)	C34—C35—C36	120.0 (7)
C19—C14—C13	122.6 (5)	C34—C35—H35	120.0
C14—C15—C16	121.7 (7)	C36—C35—H35	120.0
C14—C15—H15	119.1	C35—C36—C37	119.8 (7)
C16—C15—H15	119.1	C35—C36—H36	120.1
C17—C16—C15	120.2 (7)	C37—C36—H36	120.1
C17—C16—H16	119.9	C36—C37—C38	119.3 (7)
C15—C16—H16	119.9	C36—C37—H37	120.4
C18—C17—C16	119.0 (7)	C38—C37—H37	120.4
C18—C17—H17	120.5	C33—C38—C37	121.5 (7)
C16—C17—H17	120.5	C33—C38—H38	119.3
C17—C18—C19	120.4 (7)	C37—C38—H38	119.3
C17—C18—H18	119.8	C7—N1—C8	120.6 (4)
C19—C18—H18	119.8	C13—N2—C10	109.5 (4)
C18—C19—C14	121.8 (6)	C13—N2—C11	111.3 (4)
C18—C19—H19	119.1	C10—N2—C11	109.0 (4)
C14—C19—H19	119.1	C26—N3—C27	119.9 (4)
C21—C20—C25	118.0 (5)	C29—N4—C32	109.3 (4)
C21—C20—C26	120.4 (5)	C29—N4—C30	109.8 (4)
C25—C20—C26	121.7 (5)	C32—N4—C30	111.1 (4)
C22—C21—C20	121.7 (6)	C6—O1—H1	111 (4)
C22—C21—H21	119.2	C25—O2—H2A	109 (4)
			
C6—C1—C2—C3	1.4 (8)	C21—C20—C25—C24	0.3 (7)
C7—C1—C2—C3	−177.8 (5)	C26—C20—C25—C24	179.7 (5)
C1—C2—C3—C4	−1.0 (9)	C21—C20—C26—N3	178.2 (5)
C2—C3—C4—C5	−0.5 (9)	C25—C20—C26—N3	−1.1 (7)
C3—C4—C5—C6	1.4 (9)	N3—C27—C28—C29	174.9 (4)
C4—C5—C6—O1	178.9 (5)	C31—C27—C28—C29	54.5 (6)
C4—C5—C6—C1	−0.9 (8)	C27—C28—C29—N4	−58.7 (6)
C2—C1—C6—O1	179.7 (5)	N4—C30—C31—C27	57.2 (6)
C7—C1—C6—O1	−1.1 (7)	N3—C27—C31—C30	−173.5 (4)
C2—C1—C6—C5	−0.5 (7)	C28—C27—C31—C30	−54.0 (6)
C7—C1—C6—C5	178.7 (5)	N4—C32—C33—C38	−85.8 (7)
C2—C1—C7—N1	−179.7 (5)	N4—C32—C33—C34	96.1 (7)
C6—C1—C7—N1	1.2 (7)	C38—C33—C34—C35	−0.1 (9)
N1—C8—C9—C10	−171.3 (4)	C32—C33—C34—C35	178.0 (6)
C12—C8—C9—C10	−50.2 (6)	C33—C34—C35—C36	−1.6 (10)
C8—C9—C10—N2	55.4 (6)	C34—C35—C36—C37	2.1 (11)
N2—C11—C12—C8	−58.8 (6)	C35—C36—C37—C38	−0.9 (11)
N1—C8—C12—C11	172.8 (4)	C34—C33—C38—C37	1.4 (9)
C9—C8—C12—C11	52.1 (5)	C32—C33—C38—C37	−176.8 (5)
N2—C13—C14—C15	−136.4 (6)	C36—C37—C38—C33	−0.9 (10)
N2—C13—C14—C19	45.2 (8)	C1—C7—N1—C8	−179.5 (4)
C19—C14—C15—C16	−0.2 (10)	C9—C8—N1—C7	−118.2 (5)
C13—C14—C15—C16	−178.7 (6)	C12—C8—N1—C7	120.8 (5)
C14—C15—C16—C17	0.0 (12)	C14—C13—N2—C10	−177.4 (5)
C15—C16—C17—C18	−0.1 (12)	C14—C13—N2—C11	62.1 (6)
C16—C17—C18—C19	0.5 (12)	C9—C10—N2—C13	178.0 (4)
C17—C18—C19—C14	−0.7 (12)	C9—C10—N2—C11	−60.0 (5)
C15—C14—C19—C18	0.6 (10)	C12—C11—N2—C13	−177.6 (4)
C13—C14—C19—C18	179.0 (6)	C12—C11—N2—C10	61.6 (5)
C25—C20—C21—C22	−0.8 (9)	C20—C26—N3—C27	−178.4 (4)
C26—C20—C21—C22	179.9 (6)	C31—C27—N3—C26	−132.8 (5)
C20—C21—C22—C23	0.5 (11)	C28—C27—N3—C26	107.9 (5)
C21—C22—C23—C24	0.3 (10)	C28—C29—N4—C32	−177.8 (4)
C22—C23—C24—C25	−0.7 (9)	C28—C29—N4—C30	60.1 (5)
C23—C24—C25—O2	180.0 (5)	C33—C32—N4—C29	169.3 (5)
C23—C24—C25—C20	0.4 (8)	C33—C32—N4—C30	−69.5 (6)
C21—C20—C25—O2	−179.3 (5)	C31—C30—N4—C29	−58.7 (6)
C26—C20—C25—O2	0.1 (7)	C31—C30—N4—C32	−179.7 (4)

### Hydrogen-bond geometry (Å, °)

**Table:**

*D*—H···*A*	*D*—H	H···*A*	*D*···*A*	*D*—H···*A*
O1—H1···N1	1.01 (8)	1.73 (7)	2.597 (5)	141 (6)
O2—H2A···N3	1.05 (7)	1.66 (7)	2.588 (6)	144 (5)
O1—H1···O1^i^	1.01 (8)	2.49 (7)	2.869 (7)	102 (5)

Symmetry codes: (i) −*x*+1, −*y*+1, −*z*+2.
